# Care-deficits and polarization: Why the time is ripe for a universal care conscription

**DOI:** 10.1177/09697330211046649

**Published:** 2022-02-09

**Authors:** Bouke de Vries

**Affiliations:** Umeå University, Sweden; KU Leuven, Belgium

**Keywords:** Adolescents, aged care, care conscription, democracy, care-worker shortages, polarization, social cohesion

## Abstract

A large share of countries is struggling to provide adequate care to their older populations. To deal with this challenge, philosopher Ingrid Robeyns has advocated legislation that requires (most) citizens to spend 1 year of their life providing dependency care. My aim of this contribution is to strengthen the case for this proposal, which I will refer to as a ‘universal care conscription’. I do so by defending this type of conscription against various alternative ways of addressing care-deficits that have been proposed. As I show, not only is it doubtful whether pursuing these alternatives will always, or even generally, suffice to prevent and/or alleviate care-deficits at reasonable financial and moral cost, a universal care conscription has significant civic benefits in an age of polarization that count strongly in its favour.

## Introduction

Many countries have care-worker shortages that prevent them from providing adequate dependency care, construed broadly to include both material care and social and emotional care.^i^ For example, a study from 2012 showed that nurse staffing standards and staffing levels were lower than what experts advised in countries such as the United States, Canada, England and Germany.^2^ In a more recent study, health economist Heinz Rothgang found that German nursing home residents currently receive a daily average of 99 min of care, which falls well short of the recommended average of 141 min.^3^ When we consider that almost all countries are ageing and will continue to do so in the coming years and decades^4^ – for example, the share of Germans aged 60 years and above is projected to rise from 27% in 2014 to 35% in 2030 and to 38% in 2050^5^ – it becomes clear that these problems are unlikely to disappear in the foreseeable future and, indeed, likely to become worse, with some estimates suggesting that there will be a shortage of nearly half a million care-workers in Germany by 2030.^6^

To deal with this challenge, philosopher Ingrid Robeyns has advocated legislation that requires (most) citizens to spend 1 year of their life providing dependency care. My aim of this contribution is to strengthen the case for this proposal, which I will refer to as a ‘universal care conscription’. I do so by defending this type of conscription against various alternative ways of addressing care-deficits that have been proposed. As I show, not only is it doubtful whether pursuing these alternatives will always, or even generally, suffice to prevent and/or alleviate care deficits at reasonable financial and moral cost, a universal care conscription has significant civic benefits in an age of polarization that count strongly in its favour.

## The care-based argument for universal care conscription

In a short essay on caregiving practices within contemporary Western societies, Ingrid Robeyns calls for the ‘the implementation of a citizen’s duty to care’,^7^ which follows a similar call by Diemut Bubeck who more briefly discusses the idea of a care conscription in a paper on feminist citizenship.^8^ Under this proposal,

[A]ll citizens should, upon reaching a certain age (say, the age of advanced adolescence or adulthood), spend some time caring for those who are in need of care: either small children, the disabled, vulnerable elderly, or the ill. By imposing this as a moral and political duty on all citizens, one would make sure that all adults have had, at the start of their adult life, a significant experience of actually performing care work. The duty should be universal – that is, it should be carried out by all members of society, except if some strong reasons make those members unsuited.^7^

According to Robeyns, those who are unsuited to perform a care duty include those ‘who are somehow impaired in their abilities which are needed to care’, which, as she goes on to note, include the ‘mentally disabled’, as well as those who lack the requisite dispositions such as individuals with a ‘dominant, aggressive character’, whom she plausibly suggests should first be taught to provide care to other humans and, insofar as such educational attempts are ineffective, be required to look after animals or after a piece of nature such as a forest.^7^ In addition to this, she suggests – again plausibly – that those who have already performed a large amount of care-work prior to conscription age should either be exempted or simply be given a smaller workload.^7^

Why think that having a universal care conscription in the sense just described is morally desirable? Robeyns offers several reasons. One is that, by increasing the supply of care-workers, it allows older adults and other care-dependent groups to *receive more care*, including more social and emotional care, which is of great importance in countries with care-worker shortages, of which it was noted there are many (see the ‘Introduction’ section).^7^ Not only would citizens be providing a considerable amount of care during their conscription, one might reasonably expect that the experience will induce a proportion of them to provide more, as well as better, care than they would have provided otherwise – this is true particularly of men, as women tend to face greater social expectations to provide informal care from a young age onwards^9^ and spend, on average, more time on caregiving and housework within heterosexual co-residing relationships, especially after child birth,^10,11^ which is why a universal care conscription is believed to promote gender justice by authors such as Robeyns, Bubeck and Berges.^1,7,8^ Apart from the fact that those who complete such a conscription might learn valuable caregiving skills or simply hone their existing skills, many are likely to gain a (better) appreciation of the potential burdens and difficulties of caregiving and, as Robeyns puts it, stop seeing it as ‘merely a hobby or unskilled labour’, which she believes is likely to make them more willing to share fairly in this type of work at home and become more supportive of care-workers being properly remunerated.^1,7^ I think these are plausible conjectures and would add that, besides becoming (more) aware of its potential costs, those who complete a universal care conscription may also become more aware of the potential value of caregiving, which can be a highly meaningful and rewarding activity,^12^ and become more willing to provide care as a result.^ii^

Of course, these predictions are speculative as no country has, to the best of my knowledge, ever had a universal care conscription. However, there are countries such as Japan that have universal home economics classes for junior and senior high school students that similarly teach students how to provide care and have led to increases in the amounts of informal caregiving among couples. Analysing the impact of a 1989 educational reform during which home economics became a mandatory subject in junior and senior high school for boys born after 1977, which had previously been a mandatory subject for girls only, Hiromi Hara and Nuria Rodriıguez-Planas found that there was ‘a sharp increase [for the post-1977 cohort] in both the amount and the share of the typical husband’s household production’, particularly with respect to caregiving, as well as an increase in the overall amount of caregiving that couples performed.^13^

Having focused hitherto on the benefits of a universal care conscription for the care-recipients, it should be observed that its introduction might also benefit *existing caregivers* by reducing their workload. This group comprises both formal (i.e. paid) caregivers, who in many countries report high levels of emotional exhaustion and burnout rates,^14^ and informal (i.e. unpaid) caregivers, of whom a large share reports similar problems – for example, a 2016 survey from the Netherlands where one in seven informal caregivers, who on average provided 28 h of care a week, said that the burdens of their caregiving were either ‘heavy’ or ‘too heavy’15 – and who do not rarely find themselves in financially precarious situations due to the earnings that they forego as a result of performing this type of labour.^16^

## The less-restrictive-alternatives objection

While it seems clear then that a universal care conscription would have significant advantages in societies with current or expected care-deficits, there is an important objection to such a conscription that Robeyns does not address in her short essay. According to this objection, there are ways of preventing and alleviating care-deficits that are *morally preferable* on account of being less restrictive and therefore more respectful of people’s autonomy. These might include, but are not necessarily limited to, the following:

– Offering higher salaries to formal caregivers in order to incentivize people to work in this sector;
– Providing financial compensation and social support to informal caregivers so that more people become willing to provide (more) unpaid care;
– Recruiting caregivers from low-income countries, as many high-income countries are currently doing (think, for example, of the recruitment of Filipino nurses by countries such as Germany, the United Kingdom, and Canada);^17^
– Investing in robot care and other forms of assistive technology, such as smart tracking devices.^18,19^

Before responding to this objection, which I will refer to as the ‘less-restrictive-alternatives objection’, I should stress that those who believe that this objection counts decisively against a universal care conscription need not deny that most, if not all, alternatives to this type of conscription have problems of their own. For example, using robot care raises concerns about the privacy of care-recipients, as well as ones about the possibility of deception when care-recipients do not realize that they are interacting with a non-sentient being,^18^ whereas recruiting foreign care-workers has a tendency to create care-deficits within the sending societies,^20^ which are often ageing as well.^21^ Accordingly, rather than having to accept that there exist flawless alternatives to a universal care conscription, all that proponents of the current objection are committed to is that, on the whole, the moral costs of pursuing one or more of these alternatives are *outweighed* by the moral costs of legally requiring citizens to sacrifice 1 year of their lives providing care to others.

## Rejoinders to the less-restrictive-alternatives objection

### The potential indispensability of a care conscription

What to make of the less-restrictive-alternatives objection? Although it poses a serious challenge, I think that, in many societies, there remain decisive reasons for introducing a universal care conscription. One way of showing this is to point out that alternative forms of care-provision are unlikely to wholly eliminate substantial care-deficits or simply unlikely to do so at reasonable financial and moral cost, thereby leaving intact the need for conscripting citizens (which, of course, does not rule out that one or more alternative ways of preventing and alleviating care-deficits ought to be pursued; all it means is that the availability of such alternatives does not render a universal care conscription superfluous). Consider again the case of Germany where it was noted that a shortage of nearly half a million care-workers is expected by 2030.6 Whenever such large shortages exist, it is doubtful whether a universal care conscription can be reasonably avoided.

To bring this out, notice that seeking to wholly, or even merely largely, close such gaps by offering higher salaries to formal caregivers in order incentivize people to join and remain active in this sector is likely to be prohibitively expensive in many societies. This is not only because of the sheer number of workers that is needed, but also because the high work pressure – which is unlikely to abate any time soon due to population ageing – will require a lot of extra money to be offered in order to convince enough people to (continue to) do this type of work. While governments could try to address any remaining care-deficits by offering (more) financial compensation and other forms of support to informal caregivers, it is doubtful whether this will entirely, or even just largely, solve the problem. To see this, recall that within countries such as the Netherlands, a significant share of informal caregivers is already overwhelmed by the workload,^15^ which due to population ageing is unlikely to change within the foreseeable future even when (additional) public investments are made. (Which, to be clear, does not necessarily mean that (additional) public investments in formal and informal care should not be made; all it means is that such investments are unlikely to render a universal care conscription superfluous.)

What about the other two alternative measures that were mentioned? Even when they are implemented in tandem with the measures just mentioned, I think there are good grounds for being sceptical about their ability to wholly, or even merely largely, offset severe care-deficits at reasonable financial and moral cost. As for the recruitment of caregivers from low-income countries, it was noted that this type of migration has a tendency to generate care-deficits within the sending societies,^20^ which comes alongside a host of other problems and challenges such as the fact that it breaks up families and the fact that receiving countries will need to invest resources in the migrants’ integration within society.^22^ As for the use of robot care and other forms of assistive technology, such as smart tracking devices, what we find is that these forms of technology have not (yet) reached a stage of advancement where they can wholly replace human care. Nor would such a complete replacement be morally desirable insofar as we accept, as many philosophers do,^23–25^ that we have dignity-interests in receiving a meaningful part of our care and companionship from other humans, apart from any autonomy-interests and hedonic interests that we might have in receiving this type of care and companionship.

Admittedly, even if I am right that (1) conscripting citizens to provide care for a year will make a sizable contribution to the prevention and/or reduction of heavy care-deficits within society (see the penultimate section) and that (2) the most promising policy alternatives to a universal care conscription are unlikely to completely, or even merely predominantly, close such deficits at reasonable financial and moral cost, it does not follow that instating such a conscription must be morally justified. For this to be the case, the costs of doing so must also be *proportional* to the aims served.

I believe that this condition is satisfied within societies with large shortages of care-workers or ones where such shortages are expected within the near future. Given that receiving adequate dependency care has an enormous impact on our health and well-being, which are among the most valuable goods in life, the interests served by a universal care conscription are evidently very weighty ones and, indeed, ones that seem weighty enough to require people to spend 1 year of their lives performing this type of service. This is not simply because a single year is a relatively short period, especially within societies with a life-expectancy of around 80 years, although this is an important reason. The occupational restrictions imposed by a universal care conscription are also ones that are likely to *benefit the conscripts themselves* in various ways, if not immediately then at later stages of their lives. Let me mention four ways.

First, those who are conscripted will often (eventually) become recipients of the care provided during this type of conscription themselves, which is particularly likely to occur during the final stages of their lives when many of us come to require dependency care. Indeed, to the extent that there has been a first generation of conscripts already, people might *already have been the recipients* of said care during (early) childhood when all of us are heavily reliant on others’ care for our survival and development.

Second, most conscripts are likely to have at least some loved ones (e.g. an indigent parent, a disabled sibling, a partner with a long-term illness) who will benefit from the additional care that a universal care conscription makes available. What is important for present purposes is that when our loved ones procure such benefits, this is likely to *increase our own well-being as well* given that we generally care deeply about the fate of those dearest and nearest to us.^1^

Third, by dividing care-work more evenly among the population,25 a universal care conscription reduces the amount of informal care that many conscripts need to provide to loved ones if they want the latter to enjoy a minimally decent living standard. This too is an important benefit given that, as we have seen, informal caregiving frequently takes a heavy physical, psychological and financial toll even if it simultaneously has the potential to imbue people’s life with meaning and value. Furthermore, even when people are not legally required to perform it, there will often be social pressure on them to do so, especially on women due to the presence of gendered norms (see the penultimate section), which is also likely to wane as the need for informal caregiving is being reduced.

Fourth, doing a care conscription is likely to equip conscripts with caregiving skills or help them to improve any existing caregiving skills. To see how this benefits them, it must be observed that there is a good chance that other individuals (e.g. young children, indigent parents, ill partners) will come to rely on their care at some point insofar as they do not do so already. In fact, even when people end up making little use of these skills during their lives, having them remains valuable just to be prepared for such eventualities – one might draw a comparison with knowing how to deliver firs aid; even if one never uses one’s first aid skills, having them is still valuable given the possibility that some unfortunate person will come to rely on them in a crisis situation.

### Civic benefits

Thus far, I have defended a universal care conscription against an important neglected objection, namely the claim that there are morally preferable alternatives to such a conscription that states should pursue instead to prevent and mitigate care-deficits. What I want to do in this section is to bolster the case for a universal care conscription further by offering a new argument for it. This argument, which I will refer to as the ‘civic benefits-argument’, is predicated on the assumption that, when designed properly, a universal care conscription is likely to have a *salutary effect on a country’s civic culture*.

To explain further, it bears mentioning that many (Western) societies have become heavily polarized in recent years. In the United States, for instance, a party-ideological rift has emerged whereby 44% of Democrats and Democratic leaners now view the Republican Party very unfavourably and 45% of Republicans and Republican leaners view the Democratic Party very unfavourably, a marked difference with the mid-1990s when these rates were below 20% for both groups.^26^ Or consider the United Kingdom where a large policy-ideological division has arisen over the Brexit referendum. According to some polls, a majority of both Remain-supporters and Leave-supporters see the other side as ‘hypocritical’, ‘selfish’ and ‘closed-minded’, which is reflected in, inter alia, a widespread unwillingness to talk politics with members of the other side – only around half of each group report being happy to do this^27^. While modest levels of polarization are generally considered to be healthy for democracies as they are evidence that citizens take an active interest in politics, such high levels have been shown to be maladaptive as they severely undermine people’s willingness to collaborate with, and make concessions to, individuals from across the political aisle, which produces political paralysis and might ultimately result in democratic backsliding.^28^

To see how a universal care conscription can help to address these ills, it should be noted that studies have shown that positive intergroup contact reduces polarization by mitigating animus towards out-group members,^29–31^ which has led some authors to call for educational institutions and firms to actively facilitate such contact.^29,32,33^ However, a universal care conscription might facilitate it *as well* when conscripts are made to work in teams that comprise people with different ideological views and different backgrounds (e.g. ethnic, religious, socio-economic), especially when citizens are conscripted straight after secondary school so that the intergroup contact still takes place during their formative years. (To help ensure that such contacts are positive, I take it that states may need to, for example, organize team-building events and regularly remind conscripts of their shared national identity as superordinate identities have been found to reduce partisanship.)^34^

It might be argued that a universal military conscription can realize these civic benefits *just as effectively and efficiently*, and that this renders my case for a universal care conscription underdetermined. A first thing to say in response to this argument, call it the ‘underdetermination objection’, is that its empirical premise seems false. One reason for this is that many societies harbour citizens with conscientious and/or religious objections to serving in the military who will often deeply resent being conscripted – think, for instance, of Israel, where the Haredim have religious objections to serving in the military and where initiatives to revoke their exempt status have generated much anger among their community and sparked protests.^iii^^35^ Another reason is that there exists evidence that military service actually *amplifies civic distrust* among men. Leveraging quasi-random variation in conscription reforms across 15 European countries, a study by Bove et al. thus found that ‘cohorts that served compulsory time as soldiers have lower institutional trust than cohorts that were not exposed to military conscription’, which according to the authors is due to exposure to military norms that prompt ‘more uniformed, negative views of civil society on male draftees, at a very sensitive stage of their life’.^36^

But – and this brings me to the second response – *even if* a universal military conscription counteracted polarization just as well as a universal care conscription, having a universal care conscription would still often seem preferable. This is because within many contemporary societies, namely those where there are severe current or expected future care-deficits and where the risk of military conflict within the foreseeable future is low, people are likely to benefit more from increasing the supply of care-workers – which we saw in the penultimate section can have great value for care-recipients and caregivers alike, apart from the fact that it is likely to promote gender justice – than from increasing the supply of soldiers.

A third and final response is that even when the security and geopolitical situation are such that a universal military conscription is necessary – as it might be in countries such as Sweden and Lithuania, which have re-introduced a military conscription recently in response to Russian military aggression – this does not rule out having a universal care conscription at the same time.^25^ To be sure, showing respect for people’s autonomy might limit how many years citizens can be reasonably expected to perform these types of services, which means that each form of conscription may need to be shorter than would be morally permissible if only one of them existed. Still, the fact that such co-existence is possible, along with the fact that many societies either have severe care-deficits or will have them soon, provides yet another reason for thinking that the underdetermination objection ought to be rejected.^iv^

## Concluding remarks

This article has strengthened the case for a universal care conscription by defending it against an important neglected objection and by providing a novel argument to support it. Whereas the debunked objection maintains that there are alternatives to a universal care conscription that similarly help to prevent and/or alleviate care-deficits yet that are morally preferable on account of being less restrictive, the supporting argument maintains that such a conscription can have significant civic benefits in an age of polarization alongside its main other benefits, namely its ability to address care-deficits and to help realize gender justice.

By way of conclusion, I want to add a few clarificatory comments and suggest some avenues for future research. First, whereas I have used the term ‘universal care conscription’ throughout this article, I share Robeyns’ view that two groups of citizens ought to be partially, if not wholly, exempted. One comprises citizens who have already provided a considerable amount of informal care by the time that they are required to serve. When this is the case, it would seem unfair to expect them to do a (full) care conscription given that they have already made significant contributions to solving (future) care-deficits within society even if they did not do so with this civic aim in mind (oftentimes, they may have been simply concerned about the health and well-being of a care-dependent loved one). The other group comprises citizens who are unable or unsuited to provide care even after attending a state-sponsored training or educational programme. However, whereas Robeyns suggests that the ‘mentally disabled’ are among this group and that these individuals should be ‘freed from their citizen’s duty to care’,6 my suspicion is that many of these individuals can provide (simple) forms of care and that it is desirable that they not be exempted from this responsibility in order to treat them as full and equal citizens.^v^

Second, while the civic benefits-argument that I have offered strengthens the case for a universal care conscription within many societies, such a conscription *need not reduce polarization* in order to be justified. Specifically, it seems that, as long as there are substantial current or expected future shortages of care-workers, this will normally be enough to justify, and, indeed, morally require, a universal care conscription given the serious human costs of such deficits (see my earlier remarks). (Accordingly, even within highly cohesive societies, there might be a need for a universal care conscription.)

Third, I remain non-committal here on whether high levels of polarization within society, alongside possible unjust gender divisions in the provision of informal care, are ever enough to justify, if not morally require, a universal care conscription. Suffice it to say that, to the extent that this is the case, even a state that can prevent and/or alleviate care-deficits at reasonable financial and moral cost *without such a conscription* (as some might be able to, although this is likely to become increasingly rare as the large majority of countries worldwide are ageing) may have sufficient, if not decisive, moral reasons to introduce one.

Fourth, the arguments that I have offered for a universal care conscription are ones that can be accepted by proponents of *different ethical traditions*, including by proponents of the most prominent traditions within contemporary moral philosophy, which makes my case for this type of conscription stronger than it would be otherwise:

– Deontologists may support a universal care conscription because of the indignities and loss of freedom and autonomy suffered by people who cannot be provided with adequate dependency care;
– Utilitarians may support a universal care conscription because of the disutility suffered by this group;
– Communitarians may support a universal care conscription because of its potential to promote social cohesion and solidarity;
– Feminists may support a universal care conscription on grounds that such a conscription is likely to promote gender justice;
– Care ethicists may support a universal care conscription on grounds that this type of conscription helps to facilitate valuable caring relationships;
– Republicans may support a universal care conscription on grounds that it expresses an ideal of active citizenship and mitigates the extent to which care-dependent individuals are vulnerable to domination by making them less dependent on private charity.

Fifth and finally, there are several remaining normative issues raised by universal care conscriptions that space constraints have prevented me from addressing within this article. One was mentioned earlier in this section, namely whether the potential civic benefits of such conscriptions, along with their potential to promote gender justice, can be *enough* to justify this type of conscription within societies that are marked by polarization and/or by unjust gender divisions in caregiving. One more I address in another work, namely whether immigrants who arrive in the receiving society past the conscription age should perform this type of conscription as well in order to ensure fairness between existing members of society and newcomers, perhaps as part of the requirements for becoming permanent residents and/or citizens (Rothgang, 2020).^3^ Undergirding this issue is the worry that, *unless* such immigrants complete a care conscription too, they will benefit from publicly provided good to which they have not (directly) contributed. Still another remaining normative issue, one that also concerns a fairness question, is whether older generations who are past the conscription age when a universal care conscription is introduced should be made to perform such a conscription when they still can. In this case, the worry is that failing to do so will produce intergenerational injustice as older generations will benefit from a public service to whose provision they have not made any (direct) contributions.

My hope is that this article will inspire future work on these questions, as well as that the first governments will soon introduce a universal care conscription so that its effects can be studied empirically.

## Notes

i While it is true that such objectors could be exempted, this would also reduce the potential of this type of conscription to promote national cohesion and solidarity. For not only would there be social groups with whose members people would not interact during their service, the fact that these individuals are exempted might create bad blood among the rest of the population as the exempt status of the Haredim has done in Israel.^35^

ii Bubeck thinks that the caring dispositions and skills that are developed or honed during a universal care conscription will also make citizens more concerned about the ‘creation and maintenance of social cohesion, the general welfare of society’ and […] participation in the political sphere’, which, she goes on to write, will ‘contribute to the correction of the worst effects of the possessive and competitive individualism and atomism that has characterised modern capitalist democracies and reduced the understanding of citizenship to an exclusive focus on rights’.^8^ Since these positive ripple effects are more speculative, however, I do not want to put much weight on them here

iii While it is true that such objectors could be exempted, this would also reduce the potential of this type of conscription to promote national cohesion and solidarity. For not only would there be social groups with whose members people would not interact during their service, the fact that these individuals are exempted might create bad blood among the rest of the population as the exempt status of the Haredim has done in Israel.^35^

iv Bubeck believes that there are also *expressive* or *communicative reasons* for states to have both types of conscription. The thought here is that when a state only has a military conscription, it thereby conveys that ‘warring’ is more important than ‘caring’, which Bubeck is mistaken because, as he puts it, a society’s survival ‘depends on care for its future generations, and its flourishing depends mostly on the quality of life its citizens are able to assure not only for themselves but also for one another’.^25^

v While it might be said that when exemptions are allowed it no longer makes sense to speak of a *universal* care conscription, I believe that the use of this term remains warranted. One reason for this is that, under the proposed system, cases where people are fully exempted from this type of conscription would be relatively rare. Another reason is that the default would be for everyone to be conscripted *unless* they, or those who are legally authorised to represent them, bring forward valid reasons for wanting an exemption.
